# Structure of mycobacterial maltokinase, the missing link in the essential GlgE-pathway

**DOI:** 10.1038/srep08026

**Published:** 2015-01-26

**Authors:** Joana Fraga, Ana Maranha, Vitor Mendes, Pedro José Barbosa Pereira, Nuno Empadinhas, Sandra Macedo-Ribeiro

**Affiliations:** 1 IBMC – Instituto de Biologia Molecular e Celular, Universidade do Porto, Porto, Portugal; 2 CNC - Center for Neuroscience and Cell Biology, University of Coimbra, Coimbra, Portugal

## Abstract

A novel four-step pathway identified recently in mycobacteria channels trehalose to glycogen synthesis and is also likely involved in the biosynthesis of two other crucial polymers: intracellular methylglucose lipopolysaccharides and exposed capsular glucan. The structures of three of the intervening enzymes - GlgB, GlgE, and TreS - were recently reported, providing the first templates for rational drug design. Here we describe the structural characterization of the fourth enzyme of the pathway, mycobacterial maltokinase (Mak), uncovering a eukaryotic-like kinase (ELK) fold, similar to methylthioribose kinases and aminoglycoside phosphotransferases. The 1.15 Å structure of Mak in complex with a non-hydrolysable ATP analog reveals subtle structural rearrangements upon nucleotide binding in the cleft between the N- and the C-terminal lobes. Remarkably, this new family of ELKs has a novel N-terminal domain topologically resembling the cystatin family of protease inhibitors. By interfacing with and restraining the mobility of the phosphate-binding region of the N-terminal lobe, Mak's unusual N-terminal domain might regulate its phosphotransfer activity and represents the most likely anchoring point for TreS, the upstream enzyme in the pathway. By completing the gallery of atomic-detail models of an essential pathway, this structure opens new avenues for the rational design of alternative anti-tubercular compounds.

Glycogen is a central energy storage molecule in bacteria and the metabolic pathways associated with its biosynthesis and degradation are crucial for maintaining cellular energy homeostasis. The classical pathway for glycogen synthesis involves the enzymes GlgC-GlgA-GlgB^1^,^2^. However, a novel four-step α-glucan biosynthetic pathway (a.k.a. GlgE pathway) has been recently identified^3^,^4^. In mycobacteria, the GlgE pathway involves the combined action of trehalose synthase (TreS), maltokinase (Mak) and maltosyltransferase (GlgE)^4^,^5^,^6^ for the synthesis of linear α-1,4-glucans, which are substrates for the α-1,6 branching enzyme GlgB^7^. Although these four enzymes have been proposed to be essential in *Mycobacterium tuberculosis* by saturation mutagenesis experiments, pointing to critical roles in the pathogen, targeted disruption of the *mak* gene remains to be performed^1^,^3^,^8^,^9^. Together with TreY/TreZ, this pathway forms a cycle in which trehalose is recycled into and from glycogen^2^.

Trehalose is essential for mycobacterial survival, playing roles in cell wall assembly and as component of surface glycolipids that modulate host immune responses^10^,^11^. Mycobacteria possess three distinct pathways for trehalose synthesis: OtsA/OtsB, TreY/TreZ, and TreS^12^,^13^. Although TreS was believed to preferentially isomerize maltose to trehalose (or glycogen) with only trace activity in the reverse direction^14^,^15^, it was shown that the mycobacterial TreS converts trehalose to maltose and is dispensable for trehalose synthesis *in vivo*^6^,^9^,^12^. In the recently identified TreS-Mak-GlgE-GlgB pathway maltose is produced by TreS, phosphorylated by Mak into maltose-1-phosphate (M1P), and then used for glycogen elongation by the maltosyltransferase GlgE^5^,^6^. While M1P was originally identified in *Mycobacterium bovis* BCG cell extracts^16^, the enzyme responsible for its biosynthesis, Mak (EC 2.7.1.175), was only discovered thirty years later in members of the related genus *Actinoplanes*^17^,^18^. It has been also suggested that M1P could serve as substrate for the elongation of the important glycogen-related capsular α-glucan and intracellular methylglucose lipopolysaccharides, hypotheses still lacking experimental support^1^,^3^,^19^.

The GlgE-dependent α-glucan biosynthetic pathway was genetically validated as essential and its potential as drug target discussed extensively^3^,^5^. Novel inhibitors mimicking the GlgE substrate, M1P, were recently developed^20^. Remarkably, GlgE inhibition elicits the build-up of M1P, shown to be toxic for mycobacteria^3^. It has been proposed that metabolite flux through the GlgE pathway is strongly influenced by the ATP-driven M1P synthesis by Mak^3^,^21^, whose encoding gene (Rv0127) in *M. tuberculosis* H37Rv was also proposed to be essential for growth^8^.

The structural, biochemical and mechanistic analysis of the glycogen synthesis-associated GlgE pathway enzymes is therefore instrumental for designing new molecules with potential application in anti-tuberculosis therapies. In fact, with the notable exception of Mak, the three-dimensional structures of all other enzymes in this pathway have been determined recently^21^,^22^,^23^,^24^,^25^. The three-dimensional structures of *M. smegmatis* and *M. tuberculosis* TreS revealed a C-terminal carbohydrate-binding domain, which was proposed to be relevant for glycogen recognition and to provide anchorage of this enzyme to the site of glycogen polymerization^22^,^24^. Structural and biochemical data also suggested that TreS and Mak form a hetero-octameric complex, enhancing Mak catalytic activity in M1P biosynthesis^24^, possibly by favouring substrate channelling. This finding is in good agreement with the identification of numerous Mak orthologs in members of the actinobacteria and in distantly related phyla, where they frequently occur as trehalose synthase/maltokinase (TreS-Mak) bifunctional units (Figure 1)^2^,^26^.

The maltokinase from *Mycobacterium bovis* BCG, identical to the *M. tuberculosis* ortholog (Mak^Mtb^), was characterized in detail, identifying ATP as the preferential phosphate donor and the requirement of Mg^2+^ for maximal enzyme activity^26^. However, given the instability of Mak^Mtb^, we have expressed and purified the ortholog from *M. vanbaalenii* (Mak^Mvan^) for biochemical characterization and solved the three-dimensional structures of its free and non-hydrolysable ATP-bound forms. The structure of Mak^Mvan^, which shares 59% amino acid sequence identity with Mak^Mtb^, revealed a typical bilobal eukaryotic protein kinase-like (ELK) fold, conserving the main structural motifs required for the phosphotransfer reaction. Strikingly, Mak^Mvan^ displays a novel N-terminal domain, unique to maltokinases and conserved in the bifunctional TreS-Mak proteins, and without sequence similarity to other known proteins. This novel domain, topologically similar to protease inhibitors of the cystatin family^27^, is proposed to act as an anchoring point tethering maltokinase and trehalose isomerase activities to the site of glycogen biosynthesis, ensuring correct regulation of Mak activity and possibly preventing excessive accumulation of M1P. The maltokinase structures here described also provide the first structural insight into a subfamily of ELKs, as well as the framework for the discovery of new antimycobacterial drugs, contributing towards better solutions to one of the most insidious and re-emerging infectious diseases in the world.

## Results

### Maltokinase genomic context and phylogenetic analysis

**Figure 1:**
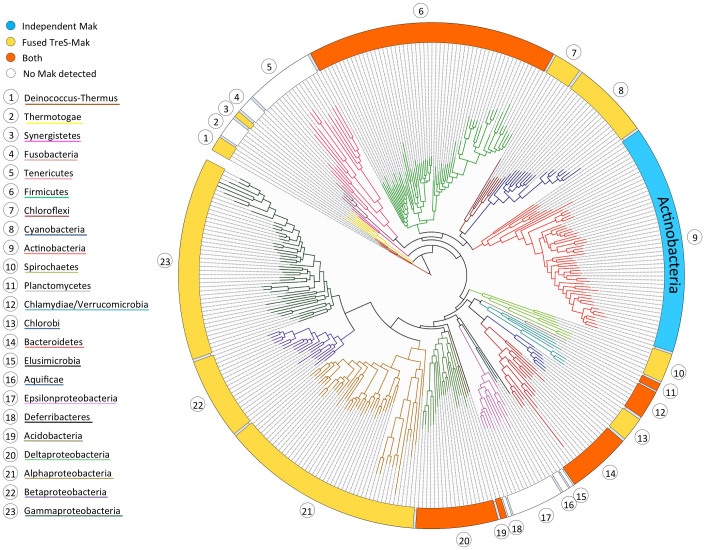
Maltokinase distribution in the bacterial domain. Maximum likelihood phylogenetic tree, built using 350 bacterial species and a concatenated alignment of 31 highly conserved protein-coding genes for phylogenetic inference (adapted by permission from Macmillan Publishers Ltd: Nature^62^, copyright 2009). Bacterial phyla and type of Mak occurrence are identified by different colours.

Putative homologs of maltokinases (EC 2.7.1.175) are present in almost all known bacterial phyla (Figure 1), as well as in some euryarchaea and crenachaea but not in the domain eukarya. Amino acid sequence identity between Mak from different taxa can be as low as 10%. Even within the *Mycobacterium* genus sequence conservation among Mak orthologs can drop below 60% (e.g. 59% between Mak^Mtb^ and Mak^Mvan^). Although several Mak proteins have now been functionally characterized, namely the enzymes from *Actinoplanes missouriensis*, *Streptomyces coelicolor*, *M. tuberculosis*/*M. bovis* BCG, *M. smegmatis* and *M. vanbaalenii* (this work), the majority of other putative Mak orthologs are still automatically annotated as aminoglycoside phosphotransferases (EC 2.7.1.95)^18^,^26^,^28^.

In the available genomes, *mak* genes are often found in the vicinity of those encoding GlgE and GlgB and in most phyla bifunctional TreS-Mak proteins are dominant (Figure 1)^2^,^26^. In mycobacteria, Mak and TreS are independent enzymes encoded, with very few exceptions, by contiguous genes, while genes encoding GlgE and GlgB, are found in distinct locations of these genomes^2^. A few mycobacteria (e.g. *M. abscessus* and *M. leprae*) lack an obvious Mak. This is not surprising for *M. leprae*, which is also devoid of most enzymes for glycogen metabolism as consequence of the genomic reductive evolution that rendered this species an obligate intracellular pathogen^29^. In fact, loss of glycogen metabolism is a common occurrence and even a marker in parasitic bacteria^30^. The reason for the absence of an obvious Mak from *M. abscessus* is still elusive^31^.

### Mak enzymatic activity and overall architecture

**Figure 2:**
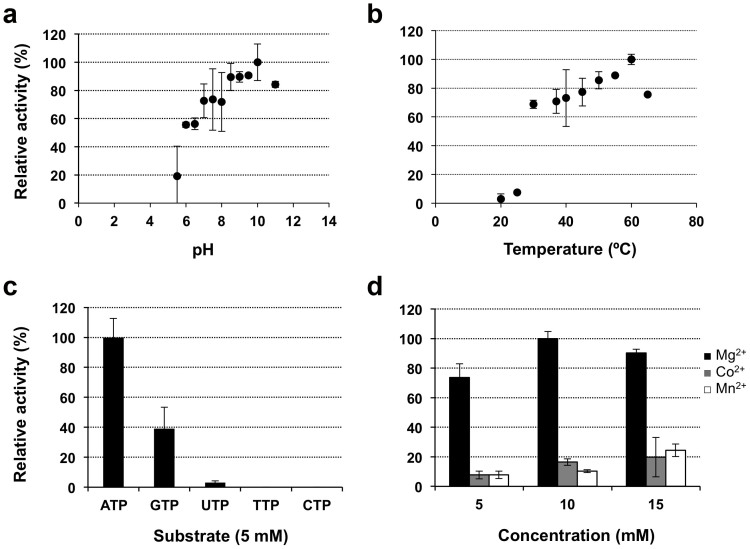
Biochemical characterization of Mak^Mvan^. Influence of buffer pH (A) and temperature (B) on Mak^Mvan^ enzymatic activity. (C) Specificity of Mak^Mvan^ for different phosphate donor substrates. (D) Effect of divalent cations on enzyme activity. Plotted data are the mean of three independent experiments.

In order to characterize the molecular details underlying maltose phosphorylation in mycobacteria, recombinant Mak^Mtb^ was produced and screened for crystallization, albeit without success. Therefore, the Mak^Mvan^ ortholog, 59% identical to Mak^Mtb^ (Supplementary Fig. S1) was selected for biochemical and structural characterization. Mak^Mvan^ displayed maltokinase activity and showed over 10-fold higher catalytic efficiency than Mak^Mtb^ (Table 1). Mak^Mvan^ used ATP as the favourite phosphate donor, being strictly dependent on divalent cations, with a clear preference for Mg^2+^ (Figure 2). Maltose was the only sugar acceptor used by Mak^Mvan^ and upon incubation with pure M1P (6 or 18 mM) no reverse reaction could be detected, even in the presence of ADP as putative acceptor. The maximal activity for Mak^Mvan^ was observed at 60°C (Figure 2), matching its melting temperature determined by DSF.

**Figure 3:**
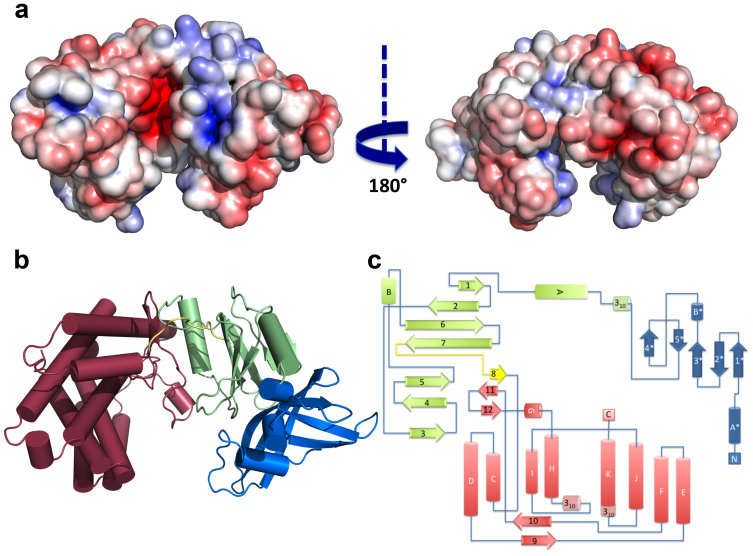
Overall structure of Mak^Mvan^. (A) Solid-surface representation of Mak^Mvan^, with mapped electrostatic surface potential contoured from +5 (blue) to −5 (red) kbTe^−1^ [kb, Boltzmann's constant; T, temperature (K); e, charge of an electron]. The left and right views are related by a 180° rotation around the vertical axis. (B) Cartoon representation of Mak^Mvan^ three-dimensional structure, coloured with the distinct domains in different colors (cap domain: blue; intermediate domain: green; C-terminal domain: pink). (C) Topology diagram of Mak^Mvan^, color-coded as in panel B. Panels A and B were prepared with PyMOL (http://www.pymol.org).

Mak^Mvan^ crystallized in the orthorhombic space group P222_1_ and diffracted to 1.47 Å resolution. The protein displays an elongated concave-shaped structure that can be divided into two half-lobes with a central narrow acidic channel (~9 Å-wide and 30 Å-long), delineated by a positively charged surface patch on the N-terminal lobe and a negatively charged region on the C-terminal lobe, and displaying basic character at its exit site (Figure 3A). The N-terminal lobe can be divided into two subdomains: a cap N-terminal subdomain comprising the first 88 amino acid residues (blue; Figure 3B, 3C) and an intermediate subdomain composed of an anti-parallel β-sheet flanked by two helices (green; Figure 3B, 3C). The C-terminal lobe is mostly α-helical (dark red; Figure 3B, 3C). While the N-terminal cap subdomain and the C-terminal lobe are predominantly acidic, the intermediate subdomain is enriched in positively charged residues (Figure 3A).

The N-terminal cap subdomain is composed of three long antiparallel β-strands (β1*β2*β3*) forming a curved β-sheet that encloses the N-terminal α-helix (αA*) and a short two-stranded β-sheet (β4*β5*) running perpendicular to the longest β-sheet axis, on its concave surface. The intermediate subdomain (residues 89–200) contains a central seven-stranded β-sheet (β1β2β6β7β5β4β3) flanked by two α-helical segments (αA and αB). A nine-residue linker (residues 201–209) containing a short β-strand (β8) connects the intermediate subdomain and the C-terminal lobe. This last domain is composed of two central 4-helical bundles, a short β-hairpin (β11β12) and a small two-stranded β-sheet (β9β10).

An interface area of ~1000 Å^2^ dominated by polar contacts, covering approximately 18% and 15% of the total solvent accessible area of the cap and intermediate subdomains, respectively, tightly interconnects the two halves of the N-terminal lobe. A small hydrophobic patch in the intermediate subdomain (e.g. Leu99, Leu102, Val109, Leu112, Phe114, Val154, Phe183 and Leu197) is partly covered by the convex surface of the central β-sheet of the cap subdomain (e.g. Leu33, Leu41, Leu56 and Val57). On both edges of the interface, strong polar contacts staple these two regions and limit interdomain flexibility. Of note is the stacking interaction between Trp187 (within the β6–β7 connecting loop) and Arg53 whose side chain forms an intradomain salt bridge with Asp43 and hydrogen bonds to Trp187 main-chain carbonyl. At the N-terminus of helix αA, the strictly conserved Asp92 forms a salt bridge with Arg59 in the central β-sheet of the cap subdomain. The invariant Arg13 forms polar contacts with the main chain carbonyls of Glu134 and Asn137 (all strictly conserved residues delineating the entrance to the central acidic channel) and the conserved Trp14 stacks with Arg152 (strictly conserved), whose side chain is involved in a salt bridge with Asp88 (strictly conserved). The extent and strength of the interactions between the two subdomains indicates that their relative motion is highly interdependent.

The interface between the N- and the C-terminal domains (~820 Å^2^) involves 12% and 7% of their total solvent accessible areas, respectively. The two domains are also highly cross-linked by a significant number of salt bridges, some of them involving the side chains of conserved amino acid residues (e.g. Glu163 from the intermediate subdomain and Arg301 and Arg335 from the C-terminal domain). Analysis of the B-factor distribution reveals that the cap subdomain has the highest values for the atomic temperature factors and the C-terminal domain the lowest, suggesting that the N-terminal lobe, in particular the cap region, is more dynamic. Some of the higher B-values correspond to residues delineating the central cleft, namely Pro64-Ala71 in the cap subdomain, Ala133-Gln135 in the intermediate subdomain, Ala204-Asn206 in the linker region and Glu326-Gly328 in the C-terminal lobe.

### Mak^Mvan^ displays a typical eukaryotic protein kinase-like fold

**Figure 4:**
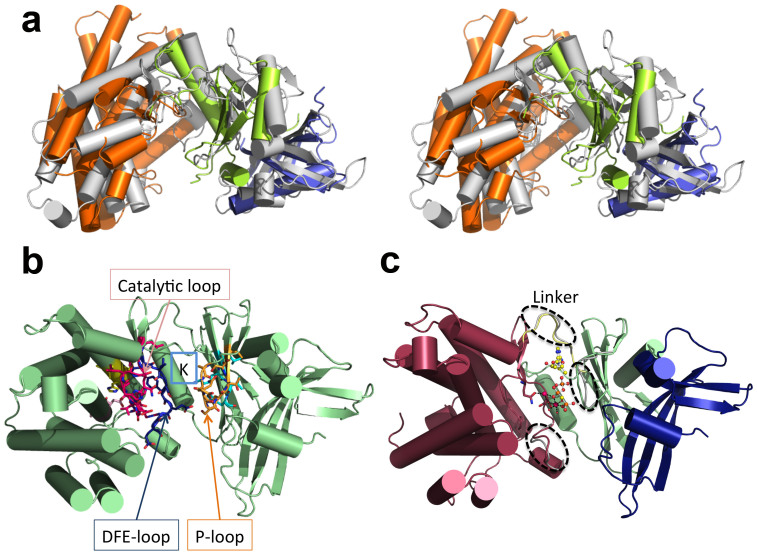
Mak^Mvan^ is structurally similar to eukaryotic protein kinase-like proteins (ELKs). (A) Stereoview of Mak^Mvan^ structure (grey cartoon) superposed with MTRK (PDB entry 2PUL^32^) N-terminal (green) and C-terminal (orange) domains, and with the cystatin domain (blue) of multicystatin (PDB entry 2W9P^63^). (B) Mapping of ELK structural motifs onto Mak^Mvan^ three-dimensional structure (P-loop: orange; AMLKV motif: cyan; catalytic loop with HGD motif: magenta; DFE motif: blue; DVA motif: light pink; VAXVH motif: yellow). (C) Cartoon representation of Mak^Mvan^:AppCp complex structure depicting the subtle structural changes required to accommodate the nucleotide (dashed ellipsoids). The bound nucleotide (for simplicity, only one of the two modelled conformations is shown) is represented as ball-and-stick (carbon: yellow; nitrogen: blue; oxygen red; phosphorous: orange), magnesium ions are shown as spheres (magenta) and the magnesium-coordinating residues (Gln310 and Asp322) are represented as sticks (atom color as in nucleotide except for carbon atoms that are coloured pink). Figures prepared with PyMOL (http://www.pymol.org).

A structural homology search using the DALI server (http://ekhidna.biocenter.helsinki.fi/dali_server/) showed that bacterial 5-methylthioribose kinase^32^ (MTRK) is the closest structural neighbour of Mak^Mvan^, despite a global amino acid sequence identity of only 7% (Figure 4A). Overall, the two structures overlap within the intermediate and C-terminal domains and superpose with an overall r.m.s.d. of 3.7 Å for 272 aligning Cα atoms (Supplementary Table S1). The closest structural neighbours identified (Supplementary Table S1) share a common protein kinase fold found in the ELK phosphotransferase family^33^,^34^. Many of the canonical structural motifs associated with nucleotide binding and enzymatic activity are conserved in Mak^Mvan^ (Supplementary Fig. S1, Figure 4B), in particular the ^146^AMLKV^150^ motif, containing the conserved phosphate-binding lysine residue; the ^241^VASVH^245^ and ^343^DVA^345^ segments, comprising the conserved histidine and aspartate residues engaged in a hydrogen bond network interlinking the nucleotide and substrate binding pockets and stabilizing the conformation of the HGD motif; the catalytic loop (^303^HGD^305^) and the ^322^DFE^324^ motif that contain the putative catalytic base (Asp305) and the magnesium-binding Asp322, respectively. This latter pattern is reminiscent of the DXD motif commonly found in glycosyltransferases that, together with the nucleotide sugar donor, coordinates the active site divalent ion^35^. As observed in related ELKs, His303 (HGD motif) stacks with Phe323 of the magnesium-coordinating DFE motif in the structure of free Mak^Mvan^. The P-loop (^133^AEQSNTSV^140^), typically containing the residues interacting with the terminal γ-phosphate of the bound nucleotide, is in Mak^Mvan^ devoid of glycine residues and is shorter than the structurally equivalent region in MTRK. As expected from the divergence in substrate specificity, the structural overlap between Mak^Mvan^ and MTRK is higher for the N-terminal lobe (intermediate subdomain) than for the substrate-binding C-terminal lobe.

A structural search with only the first 88 amino acid residues of Mak^Mvan^, corresponding to the unique N-terminal cap subdomain of maltokinases, unveiled an unforeseen resemblance with proteins displaying the cystatin fold and a remote similarity with the N-terminal domain of the serine/threonine protein kinase GCN2 (Supplementary Table S1).

### Mak^Mvan^ nucleotide binding site

The structure of Mak^Mvan^ in complex with the non-hydrolysable ATP analog, adenosine-5′-[(β,γ)-methyleno] triphosphate (AppCp), was determined at 1.15 Å resolution. The nucleotide binds with two alternative conformations within the deep pocket between the intermediate subdomain and the C-terminal domain (Figure 4C, Supplementary Fig. S3). The two conformations of AppCp overlap almost perfectly at the adenine, ribose, and γ-phosphate moieties, with the largest deviation (1.8 Å) occurring at the α-phosphate. Notably, when compared to the structurally related ELKs – e.g. MTRK and aminoglycoside phosphotransferases - the orientation of the γ-phosphate of the bound nucleotide analogs is different and directed away from the HGD loop and ~8 Å from the invariant catalytic base (Asp305; Supplementary Fig. S2).

**Figure 5:**
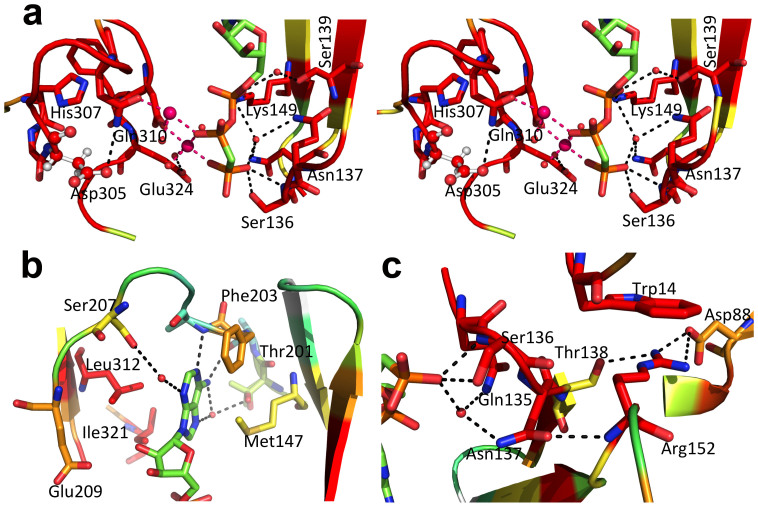
Close view of the AppCp-binding site of Mak^Mvan^. (A) Stereo view of the polar interactions centred on the phosphoryl moieties of AppCp. For all panels, residues are coloured according to conservation as shown in Supplementary Fig. S1, where red corresponds to positions strictly conserved in the actinobacteria maltokinase sequences selected for the multiple alignment. Selected residues are shown as sticks with oxygen atoms in red, nitrogen in blue, sulfur in yellow, phosphorus in orange and carbon in green (nucleotide) or according to sequence conservation (protein). The putative catalytic base (Asp305) is represented as ball-and-stick. Hydrogen bonds are represented as dashed black lines. The magnesium ions are represented as magenta spheres and dashed magenta lines represent bonds to the magnesium coordinating atoms. Red spheres represent ordered water molecules. (B) Detailed view of the interactions with the adenine base of the nucleotide bound to the active site cleft of Mak^Mvan^. (C) Close-up of the vicinity of residue Asn137, which bridges the P-loop and the terminal phosphoryl moiety of AppCp to the N-terminal cap domain through the conserved Arg152. Figure prepared with PyMOL (http://www.pymol.org).

Only local structural changes occur to accommodate the AppCp molecule, namely in the linker between the intermediate and the C-terminal domains, in the region preceding α-helix G, and in the β3-β4 connecting loop in the intermediate domain. The latter represents the most significant conformational change, where the side chain of Glu134 suffers a 180° rotation away from the negatively charged phosphate groups of the incoming nucleotide. Concomitantly, the Gln135 side chain rotates 180° to establish a water-mediated contact with the γ-phosphate group. On the opposite side, the side chain of Asp322 shifts to coordinate the magnesium ions. Two magnesium-binding sites are found in the Mak^Mvan^:AppCp complex: on the first one, also found in the free Mak^Mvan^ structure, the cation (Mg1) binds to the side chains of Gln310 and Asp322 and to the α-phosphate (in one of the conformations of AppCp); on the second site, the ion (Mg2) binds to Asp322 and bridges the β- and γ-phosphates (Figure 5A). The Glu324 residue (DFE motif) does not participate in magnesium coordination, but shifts to interact with the Mg2-coordinating water molecule in the Mak^Mvan^:AppCp complex structure.

The nucleotide establishes few direct interactions with the residues lining the enzyme's active site. The adenine group slots into a hydrophobic pocket, hydrogen bonding to the main chain of Ala202 and Ala204 (direct interactions) and to the side chains of Thr201 and Ser207 (water-mediated contacts; Figure 5B). In the AppCp complex the ribose moiety does not interact with the protein, although one of the hydroxyl groups is 4 Å away from the side-chain of the conserved Glu209. The side chain of the invariant Lys149 interacts directly with the α-phosphate and stabilizes a magnesium-bound (Mg2) water molecule. Finally, the γ-phosphate is stabilized by a direct interaction between the P-loop residue Ser136 and by water-mediated contacts to Asn137 and Lys149 (Figure 5C). In the Mak^Mvan^:AppCp complex structure the phosphate groups remain largely exposed, in contrast to eukaryotic protein kinases and MTRK, where the P-loop folds over the nucleotide, hindering solvent access (reviewed in^33^,^34^). This could partly explain why there is no interpretable electron density for the two terminal nucleotide phosphates in the Mak^Mvan^:ATP complex (Supplementary Fig. S3), maybe in result of hydrolysis during crystallization. The structure of the MakMvan nucleotide binding cleft in the ATP complex is closer to that of the apo form of the enzyme and, while the adenine moiety overlaps well with that of the nucleotide analog in the MakMvan:AppCp complex, the α-phosphate seems to be highly flexible, as revealed by the high B-factors for this portion of the molecule.

Overall, there is conservation of the residues within the nucleotide-binding site in actinobacteria, in particular those establishing either direct or water-mediated contacts with the phosphate groups on both sides of the binding cleft.

### The maltose-binding site of maltokinase

**Figure 6:**
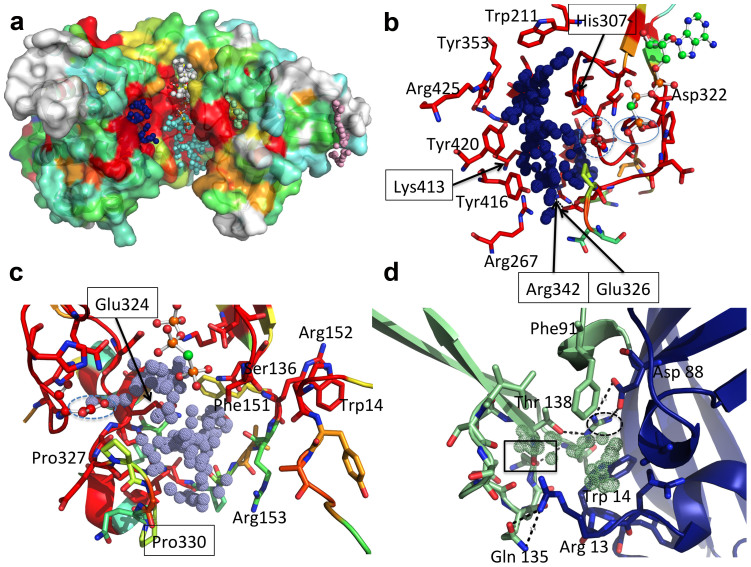
Identification of surface cavities in Mak^Mvan^. (A) Surface representation of free Mak^Mvan^ coloured according to residue conservation (consistent with the multiple sequence alignment shown in Supplementary Fig. S1). Cavities identified with the software fpocket^36^ and mentioned in the main text are represented as dotted spheres (pocket 1: blue; pocket 2: cyan; pocket 3: white; pocket 4: light green; pocket 5: light pink). (B) Close view of the residues lining pocket 1 coloured as in panel A. Residue Asp305 is represented as ball-and-stick and highlighted by a dashed blue line; the γ-phosphate of AppCp (ball-and-stick representation) is highlighted by a blue line. (C) Detailed view of residues delimiting pocket 2, coloured as in panel A; Asp305 is represented as ball-and-stick and circled by a dashed line. (D) Close view of the cavity located at the interface between the intermediate (green) and the N-terminal cap domain (blue); Asn137 is highlighted by a rectangle and Arg152 by a dashed ellipse. Hydrogen bonds are represented as dashed lines. For panels B–D, important residues are shown as sticks with oxygen atoms in red, nitrogen in blue, sulfur in yellow, phosphorus in orange and carbon in green (nucleotide) or according to sequence conservation (protein). Panels C–D refer to the structure of Mak^Mvan^ in complex with AppCp. Figure prepared with PyMOL (http://www.pymol.org).

Despite repeated attempts, no diffracting crystals of Mak^Mvan^ in complex with its sugar substrate could be obtained. In order to identify the maltose binding site, a search for suitable cavities was performed with fpocket^36^. In the structure of free Mak^Mvan^ the largest pocket identified (pocket 1, Supplementary Table S2) is located to the left of the exposed catalytic cleft and delineated by strictly conserved residues within the C-terminal lobe (Figure 6A, dark blue spheres). Pocket 1 is close to the active site HGD motif and includes Asp305, topologically equivalent to the catalytic base in analogous enzymes (e.g. MTR^32^), and globally coincident with the substrate-binding region in structurally related ELKs (Supplementary Fig. S2). This putative substrate-binding pocket, predominantly hydrophobic and with an overall positive charge, is delineated by a number of invariant residues (Figure 6B), including also Asp305 and His307 from the catalytic loop region. At the bottom of the pocket, two conserved basic residues, Arg267 and Arg342 (salt bridged to Glu326), likely direct the negatively charged reaction product away from the active site. Two additional large pockets can be identified in the vicinity of the enzyme's active site (Supplementary Table S2, Figure 6A). One of them corresponds to the nucleotide-binding site (pocket 3; Supplementary Table S2). The other pocket (pocket 2; Supplementary Table S2, cyan spheres in Figure 6A, 6C), also large enough for accommodating maltose, encompasses the γ-phosphate binding region and is framed by residues from the loop linking strand β5 and helix αB, from the P-loop and from the DFE motif–containing loop (Figure 6C). However, considering the overall higher conservation of pocket 1 in Mak^Mvan^ three-dimensional structure, which is topologically equivalent to the substrate-binding region in other ELKs, this cavity most likely represents the maltose-binding site. Accordingly, mutations of the invariant aromatic residues Tyr416 and Tyr420, and the basic residue Lys413 practically abolished maltokinase activity (Figure 5B, Supplementary Fig. S6). The observation that mutation of residues 416 and 420 to phenylalanine was sufficient to drastically reduce Mak^Mvan^ catalytic activity suggests that maltose binding involves the establishment of polar interactions with Tyr416 and Tyr420 side chains. This is in agreement with the findings in a dimeric maltokinase in complex with maltose, reported during revision of this manuscript^37^.

### Conservation of key active site residues in *M. tuberculosis* Mak

Mak^Mtb^ shares 59% amino acid sequence identity with Mak^Mvan^, with conservation of most residues within the active site cleft (Supplementary Fig. S1, S4). In the active site most differences can be identified at the adenine base-binding site, namely in the linker region and in strand β5. Relevant differences are Ile155 (Met147 in Mak^Mvan^), Glu213 and Glu215 (Ala202 and Ala204 in Mak^Mvan^), and Ala218 (Ser207 in Mak^Mvan^). Variations in this region were previously correlated with nucleoside recognition specificity in aminoglycoside phosphotransferases^38^ and could justify the experimentally observed small differences in nucleotide base specificity between these two mycobacterial enzymes (Table 1).

Overall the P-loop, the catalytic loop, and the DFE motif remain largely conserved (Supplementary Fig. S1), as well as the residues forming the identified maltose-binding pocket (Supplementary Fig. S1, Figure 6B). In fact, mutation of the invariant magnesium-binding Asp339 of Mak^Mtb^ (Asp322 in Mak^Mvan^) to asparagine completely abolished the enzymatic activity, while the mutation of the γ-phosphate interacting residue Ser144 (Ser136 in Mak^Mvan^) drastically reduced maltose phosphorylation (Supplementary Fig. S5, S7). Also, destabilization of the DFE-loop conformation, by mutating the Glu340-stabilizing (Glu324 in Mak^Mvan^) arginine side chain (mutant R351A; Arg334 in Mak^Mvan^) resulted in decreased activity (Supplementary Fig. S5, S7). Taken together, this mutational analysis underscores the overall conservation of the main structural features observed in Mak^Mvan^ in the *M. tuberculosis* enzyme.

The relevance of the tight interaction between the γ-phosphate binding loop and the cap subdomain is highlighted by the finding that mutation of Asn145 (Asn137 in Mak^Mvan^) completely inactivated Mak^Mtb^ (Supplementary Figs. S5, S7). This asparagine residue does not interact directly with the nucleotide in the Mak^Mvan^:AppCp complex, but stabilizes an ordered solvent network interlinking the P-loop, the nucleotide, and the DFE motif and helps maintaining the main-chain conformation of Arg160 (Arg152 in Mak^Mvan^), a crucial residue at the interface between the intermediate and the cap subdomains that stacks with the invariant Trp14 (Supplementary Fig. S7). These invariant residues, tightly cross-linked to the γ-phosphate binding P-loop, form part of one of the small cavities (pocket 4, Supplementary Table S2) identified in the structure of Mak^Mvan^ (Figure 6D). This conserved interface region is stabilized predominantly by a network of polar interactions, suggesting that structural changes in the flexible N-terminal cap subdomain could be easily conveyed to the nucleotide-binding region, including the P-loop, and interfere with phosphate donor stabilization and phosphoryl group transfer.

## Discussion

Many Mak orthologs have been annotated as putative aminoglycoside phosphotransferases (EC 2.7.1.95) but functional evidence is missing for most of those proteins^26^. Mak from *M. tuberculosis* is a bona fide maltokinase^26^ and recently this activity was also confirmed for the *M. smegmatis* ortholog^24^. Here we demonstrate that the *M. vanbaalenii* Mak ortholog is a maltokinase and the novel structure of Mak^Mvan^ reveals the molecular details of this new family of enzymes, identifying the conserved structural motifs associated with its phosphotransferase activity. Despite low sequence identity with aminoglycoside phosphotransferases, maltokinases display significant structural homology and conserve the main eukaryotic protein kinase-like (ELK) motifs^28^,^33^,^34^.

Maltokinase presents a bilobal arrangement with an exposed catalytic cleft. In contrast to ELKs, the N-terminal lobe has an additional N-terminal subdomain, the cap subdomain, which is unique to maltokinases. Structural analysis of free Mak and of the Mak:AppCp complex suggests that unlike protein kinases, no large lobe movements occur upon nucleotide binding, with the remarkable exception of the ample rotation of Glu134 and Gln135 side chains in the P-loop. The absence of significant interdomain motions following nucleotide attachment could (i) result from constraints imposed by crystal packing (Supplementary Fig. S8), or (ii) represent a particular conformation specific to the interaction of the ATP analog, AppCp, whose binding mode to Mak^Mvan^ might diverge from that of ATP^39^,^40^. However, the lack of significant conformational changes upon phosphate donor binding is a common feature in the structurally related ELKs such as MTRK^32^ and aminoglycoside phosphotransferases^38^,^41^,^42^, often explained by the recurrent observation of few direct interactions between the ELKs and bound ATP^43^ (PDB entry 3HAV) or non-hydrolyzable ATP analogs^32^,^41^. In particular, the absence of polar interactions between the ribosyl moiety of the nucleotide and the protein is also observed in MTRK^32^ and aminoglycoside 2″-phosphotransferase-IIa^43^, a feature previously proposed to prevent nucleotide-induced intra-lobe motion^32^.

The P-loop (a.k.a. nucleotide-positioning loop) is the structural equivalent of the glycine-rich G-loop found in eukaryotic protein kinases and MTRK, and anchors the terminal phosphate groups of the nucleotide^34^. Curiously, in the Mak^Mvan^:AppCp complex structure the γ-phosphate does not seem to be ideally oriented for phosphoryl transfer as it faces away from the putative catalytic base (Asp305) and the proposed maltose-binding pocket (Figure 5, Supplementary Fig. S2). Although it cannot be excluded that this unexpected conformation of AppCp could differ from the ATP-bound state of Mak^Mvan^, in the structurally related ELKs the terminal phosphate groups of the non-hydrolysable ATP analogs (AppNp in aminoglycoside hydrolases and AppCp in MTRK) are pointing towards the catalytic base (Supplementary Fig. S2), hinting that restrains imposed by the geometry of the non-hydrolysable analogs^39^,^40^ might not be the key reason for the observed AppCp binding mode in Mak^Mvan^. Further, the relevance of the observed interaction between the γ-phosphate and the invariant Ser136 in the P-loop is underscored by the 97% decrease of enzymatic activity upon mutation of this residue to alanine. We propose that repositioning of the terminal phosphoryl group is required for optimum phosphate transfer to the maltose acceptor, and that either substrate binding or a conformational change in the proximity of the P-loop would poise the terminal phosphoryl group for catalysis. In the current structure, the full range of this conformational alteration could be hindered by the extent of the crystal contacts on both poles of the molecule (Supplementary Fig. S8).

Conservation of the cap subdomain in maltokinases (including the bifunctional TreS-Mak enzymes), in particular of the residues in the proximity of the P-loop, together with the potential flexibility of this region as indicated by its high B-values in the Mak^Mvan^ structure, are compatible with regulatory functions for this subdomain. In fact, regulatory non-catalytic domains have been identified in eukaryotic protein kinases, modulating enzyme activity through interaction with other macromolecules^44^,^45^. It has been recently observed that *M. smegmatis* TreS directly interacts with maltokinase stimulating its enzymatic activity^24^. Considering that in many species Mak is fused to the C-terminus of TreS, suggesting spatial proximity between the N-terminal region of Mak and the C-terminal carbohydrate-binding domain of TreS^22^, it is possible to hypothesize that the N-terminal cap subdomain plays a central role in modulation of Mak enzymatic activity. In fact, a narrow pocket that could accommodate an extended molecule (e.g. a linear peptide) can be identified in the cap subdomain (pink spheres, Figure 6A). Macromolecular interactions in this region could result in a concerted motion at the interface between the cap and the intermediate subdomains, particularly affecting the dynamics of the phosphate-binding P-loop, and providing a possible explanation for the observed activation of maltokinase activity upon binding to TreS. Although no TreS homolog could so far be identified in the genome of *M. vanbaalenii*, sequence homology searches pinpointed a gene coding for a non-homologous putative TreS-like protein, Mvan_5178. In this candidate TreS, whose functional relevance in this process remains to be elucidated, relevant catalytic residues are conserved despite low overall amino acid sequence identity.

A regulated activation of maltokinase activity is in agreement with the fact that M1P is highly toxic to the cell and its production needs to be tightly controlled. In a possible model for the flow of substrates for α-glucan biosynthesis, TreS is recruited to the appropriate reservoir of glucose (glycogen) through its C-terminal domain. Upon binding to this site TreS produces maltose and associates with Mak, thereby activating the synthesis of M1P, itself channelled through GlgE for glycogen extension. The selection of the pathway leading to glycogen degradation or biosynthesis could be crucial for homeostasis of the cellular energy levels in mycobacteria and it is tempting to speculate that the enzymes involved in these pathways are associated in multifunctional protein complexes.

Maltokinase proposed essentiality in *M. tuberculosis*^8^ was hypothesized to stem from a constitutive regulatory role in sugar metabolism, in line with similar suggestions for *E. coli*^26^,^46^. The structural characterization and the identification of the molecular features associated with substrate recognition and catalytic activity of the enzymes in the essential GlgE pathway is crucial for the rational design of novel specific antimicrobial compounds. Although GlgE is a validated target for the design of specific inhibitors such as M1P analogs, a maltose transporter is lacking in *M. tuberculosis* presumably hampering the uptake of maltose analogs^21^. This argues in favour of designing pro-drugs that can be enzymatically converted by endogenous TreS and/or Mak into the biologically active GlgE inhibitors^11^,^47^. The high-resolution three-dimensional structure of mycobaterial maltokinase is a cornerstone in the structural roadmap of the essential GlgE pathway and completes the necessary experimental framework for the rational design of mycobacterial-targeting compounds that may act as narrow spectrum antibiotics^47^.

## Methods

### Sequence analysis and phylogenetic tree

Mak sequences were obtained from the NCBI database using the *M. tuberculosis* sequence (GenPept accession NP_214641.1) as template. Genome context analyses were performed with PATRIC (http://patricbrc.org/portal/portal/patric) and KEGG (http://www.genome.jp/kegg) databases. Sequence alignments were generated with T-COFFEE (http://tcoffee.grg.cat) and manually curated in MEGA6^48^. The phylogenetic tree was constructed based on a Maximum Likelihood algorithm with MEGA6 using the most appropriate model of amino acid substitution assessed by PROTTEST 3.4 and MEGA6^48^,^49^.

### Strains and culture conditions

*Mycobacterium vanbaalenii* PYR-1 (DSM 7251), obtained from the Deutsche Sammlung von Mikroorganismen und Zellkulturen GmbH (Germany), were cultivated in agar plates for 5 days at 35°C in glycerol-based medium at pH 7.0 (20 g/L glycerol, 5 g/L casaminoacids (Difco), 1 g/L fumaric acid, 1 g/L K_2_HPO_4_, 0.3 g/L MgSO_4_, 0.02 g/L FeSO_4_, 2 g/L Tween 80).

### Identification and cloning of the maltokinase gene (*mak*)

Sequence similarity searches with the amino acid sequence of *M. bovis* BCG maltokinase^26^ against the NCBI database allowed to identify the *M. vanbaalenii* maltokinase (Mak^Mvan^) sequence. The corresponding *mak* gene (Mvan_5735) was amplified by PCR from *M. vanbaalenii* chromosomal DNA using the MakF and MakR primers (Supplementary Table S3) and cloned between the NdeI and HindIII sites of pET30a.

### Site-directed mutagenesis of *M. tuberculosis* Mak (Mak^Mtb^)

The N145A and S144A *mak* mutant genes were generated with a two-round megaprimer PCR-based approach as previously described^50^. The first amplification round was performed with primers A/B1 or A/B2 or C1/D or C2/D (Supplementary Table S3) using the wild-type *M. bovis* BCG *mak* as template for insertion of each independent mutation^26^. The purified amplification products were used as templates in the second round of PCR with primers A/D (Supplementary Table S3). The final products carrying the desired mutations were cloned into pET30a, sequenced (LGC Genomics) and transformed into *E. coli* BL21 for expression.

The D339N and R351A mutants were generated by inserting a synthetic 751-base pair fragment (GenScript) carrying the desired mutation into the natural BsiWI and HindIII restriction sites of the pET30a-based construct carrying wild-type *M. tuberculosis* *mak*.

Site-directed mutagenesis with the QuikChange II Site-Directed Mutagenesis Kit (Agilent) was used to generate Mak^Mvan^ mutants K413A, Y416A, Y416F, Y420A and Y420F. Oligonucleotides used as PCR primers are listed in Supplementary Table S3.

### Protein production and purification

Recombinant wild-type and mutant Mak^Mvan^ and Mak^Mtb^ mutants were overexpressed as previously described^26^. Recombinant proteins were purified in a HisPrep FF 16/10 column (GE Healthcare) equilibrated with 20 mM sodium phosphate pH 7.4, 0.5 M NaCl, 20 mM imidazole and eluted with 200 mM imidazole in the same buffer. The purity of the fractions was evaluated by SDS-PAGE. The purest active fractions were pooled, diluted 10-fold with 20 mM Bis-Tris Propane (BTP) pH 7.4, loaded onto a 6 mL Resource Q column (GE Healthcare) equilibrated with the same buffer and eluted with a NaCl linear gradient (0–500 mM). Mak-containing fractions with highest activity and purity, as assessed by SDS-PAGE and enzymatic assays, were pooled and concentrated in 30 kDa cut-off ultrafiltration devices (Amicon) with concomitant buffer exchange for 50 mM BTP pH 7.4, 50 mM NaCl. The selected fractions correspond to monomeric enzyme as judged by size exclusion chromatography on a Superdex 12 10/300 GL column (GE Healthcare). Mak activity was monitored during purification by thin layer chromatography (TLC) using as standard maltose-1-phosphate (M1P) synthesized with recombinant Mak^Mvan^ and purified from preparative TLC silica plates. Reaction mixtures (50 μL) containing 25 μL cell-free extract or 15 μL of each chromatography fraction in 50 mM BTP pH 8.0, 3 mM ATP, 5 mM maltose, 10 mM MgCl_2_ were incubated at 37°C for 15 min prior to separation by TLC, developed with acetic acid/ethyl acetate/water/25% ammonia (6:6:2:1) and stained with α-naphthol, followed by charring at 120°C^51^. Cell-free extracts from *E. coli* BL21 carrying an empty pET30a vector were used as negative controls.

### Characterization of Mak^Mvan^ and Mak^Mtb^ mutants

Biochemical and kinetic data of Mak^Mvan^ and the kinetic parameters for Mak^Mtb^ mutants were determined as previously described^26^. Temperature and pH profiles, effect of cations and substrate specificity were determined by addition of 0.25 μg Mak^Mvan^ to 50 μL mixtures containing the appropriate buffer, 5 mM maltose, 3 mM NTP, 5–15 mM divalent cation. Reactions were stopped by cooling on an ethanol-ice bath (−10°C), followed by Mak inactivation with 5 μL 5 N HCl and neutralization with 5 μL 5 N NaOH. Controls were performed to account for NTP degradation following acid treatment. The amount of NDP released was determined by measuring the absorption at 340 nm after incubation of the sample with 3 U each of pyruvate kinase and lactate dehydrogenase, 0.3 mM NADH, 2.5 mM phosphoenolpyruvate for 10 min at 30°C^52^. ATP, CTP, GTP, TTP and UTP were tested as phosphate donors, glucose, trehalose, maltose, maltotriose, and maltotetraose as sugar substrate acceptors, and M1P was also tested as substrate for the Mak reverse reaction. The temperature profile was determined between 20 and 65°C in 50 mM BTP pH 7.5, 10 mM MgCl_2_. The effect of pH was determined at 30°C in 50 mM BTP (pH 6.0–9.0) and in 50 mM CAPS (pH 9.0–11.0), with 10 mM MgCl_2_. The effect of cations was examined by incubating the reaction mixture with the appropriate substrates and the chloride salts of Mg^2+^, Mn^2+^ or Co^2+^ at 30°C. Kinetic parameters for *M. vanbaalenii* Mak were calculated with Prism 5 (GraphPad). The *K*_M_ values for ATP, GTP, and maltose were determined at 30°C and pH 7.5. *K*_M_ and *V_max_* values for Mak^Mtb^ mutants were determined for maltose and ATP at 37°C and pH 8.0. All experiments were performed in triplicate. The specific activity of Mak^Mvan^ mutants was determined at 30°C by measuring the amount of ADP released as described above and expressed as percentage of wild-type Mak^Mvan^ maximal activity. Reactions were carried out in mixtures containing 0.25 μg protein, 5 mM ATP and 20 mM maltose in 20 mM BTP pH 8.0, 10 mM MgCl_2_.

### Differential Scanning Fluorimetry

Differential scanning fluorimetry (DSF) assays were carried out in 96-well plates on an iQ5 Optical System (Bio-Rad). Reactions contained 4 μM protein and 5x Sypro Orange dye (Invitrogen) in 50 mM BTP pH 7.5, 50 mM NaCl, 1 mM MgCl_2_. The melting curves (fluorescence emission at 570 nm, excitation at 470 nm^53^) were obtained by increasing the temperature from 25 to 85°C in 0.5°C steps with 30 s hold time and were analysed with CFX Manager software (Bio-Rad).

### Crystallization

Initial crystallization conditions in sitting-drop geometry were identified at 20°C from drops composed of identical volumes (1 μL) of protein (20 mg/mL in 50 mM BTP pH 7.5, 50 mM NaCl) and precipitant solution, equilibrated against a 300 μl reservoir. A single crystal appeared with 0.1 M MOPS/sodium HEPES pH 7.5, 0.12 M ethylene glycols (0.03 M each of di-, tri-, tetra-, and penta-ethyleneglycol), 30% PEG 500 MME/PEG 20 K as precipitant. Additional crystals were obtained by seeding techniques, using the small crystals that appeared in the crystallization drop after harvesting the original crystal. A crystal seed stock was prepared by processing the small crystals in 50 μL crystallization solution with the Seed Bead kit (Hampton Research), followed by dilution with crystallization solution until yielding single crystals^54^. The best crystals were obtained using 1 μL protein solution, 0.7 μL precipitant solution (as above, except for 28% PEG 500 MME/PEG 20 K) and 0.3 μl seed beads. The mercury derivative was prepared by soaking native crystals for 2–4 h with HgCl_2_ (5 mg/mL final concentration). Crystals of Mak^Mvan^ in complex with AppCp (adenosine-5′-[(β,γ)-methyleno] triphosphate; Jena Bioscience) or ATP were obtained from protein pre-incubated at 4°C for 1 hour with 4 mM AppCp or 1 mM ATP, respectively, and using the crystallization conditions of free Mak^Mvan^, with addition of crystal seeds. All attempts to obtain crystals of Mak^Mvan^ in complex with maltose, either by soaking or by co-crystallization were unsuccessful. Crystals of Mak^Mvan^ in the presence of maltose were also obtained with 0.1 M HEPES pH 7.5, 4.3 M NaCl as precipitant, but yielded no useful diffraction data. All crystals were cryocooled by plunging in liquid nitrogen and were stored under cryogenic conditions until data collection.

### Data collection and processing

*Mycobacterium vanbaalenii* Mak crystallized in space group P222_1_ and complete X-ray diffraction data sets were collected from single cryocooled (100 K) crystals at ESRF (Grenoble) beam lines ID14-EH1 (apo-Mak^Mvan^) and ID29 (mercury derivative and Mak^Mvan^:ATP complex), and at SOLEIL (Paris) beam line PROXIMA1 (Mak^Mvan^:AppCp complex). The apo-Mak^Mvan^ data (220 images in 1° oscillation steps, 18 s exposure per frame) were recorded on a Quantum 210 CCD detector (ADSC) using a wavelength of 0.934 Å. The mercury derivative (1400 images in 0.1° oscillation steps, 0.1 s exposure per frame), Mak^Mvan^:AppCp complex (600 images in 0.2° oscillation steps, 0.2 s exposure per frame), and Mak^Mvan^:ATP complex (1300 images in 0.1° oscillation steps, 0.037 s exposure per frame) data were recorded on Pilatus 6 M detectors (Dectris) using wavelengths of 0.976 Å, 0.918 Å, and 0.992 Å, respectively. All diffraction data were integrated with XDS^55^ and reduced with utilities from the CCP4 program suite^56^ (statistics summarized in Table 2).

### Structure determination, model building and refinement

The structure of apo-Mak^Mvan^ was solved by SIRAS with the SHELXC/SHELXD/SHELXE pipeline^57^ using data from a mercury derivative. Subsequent automated model building with ARP/wARP^58^ docked in sequence 448 out of 454 residues. The structures of the Mak^Mvan^:AppCp and Mak^Mvan^:ATP complexes were solved by molecular replacement with PHASER^59^ using the refined apo-Mak^Mvan^ structure as search model.

The models were iteratively improved in alternating cycles of manual model building with COOT^60^ and of refinement with PHENIX^61^ (statistics summarized in Table 2). The final models comprise residues Thr2-Gly441 from Mak^Mvan^ and 11 additional residues from the linker and C-terminal His-tag. Refined coordinates and structure factors were deposited at the PDB with accession numbers 4U94 (apo-Mak^Mvan^), 4U98 (Mak^Mvan^:AppCp complex), and 4WZY (Mak^Mvan^:ATP complex).

## Author Contributions

J.F., A.M., V.M., P.J.B.P. and S.M.-R. performed experiments. All authors designed research and analysed data. P.J.B.P., N.E. and S.M.-R. wrote the manuscript with contributions from all authors. All authors reviewed and approved the manuscript.

## Supplementary Material

Supplementary Information Supplementary material

## Figures and Tables

**Table 1:** Kinetic parameters of recombinant Mak^Mvan^ and Mak^Mtb^

		Maltose	ATP	GTP	UTP
	Enzyme	(with 5 mM ATP)	(with 20 mM Maltose)		
***K*_M _(mM)**	Mak^Mtb^*	2.5 ± 0.4	0.7 ± 0.1	1.0 ± 0.2	1.3 ± 0.1
	Mak^Mvan^	7.3 ± 0.9	2.6 ± 0.5	5.8 ± 1.4	ND
***V*_max_ (μmol/min.mg)**	Mak^Mtb^*	21 ± 1	21 ± 1	19 ± 1	7.1 ± 0.3
	Mak^Mvan^	301 ± 14	209 ± 19	62 ± 8	ND
***K*_cat_ (min ^−1^)**	Mak^Mtb^*	52 ± 3	54 ± 2	ND	ND
	Mak^Mvan^	3006 ± 139	2091 ± 188	616 ± 78	ND
***K*_cat_/*K*_M_ (mM ^−1^.min^−1^)**	Mak^Mtb^*	21 ± 4	72 ± 12	ND	ND
	Mak^Mvan^	414 ± 54	810 ± 163	105 ± 28	ND

*Values from the identical Mak from *M. bovis* BCG^26^.

ND - Not determined.

**Table 2:** Data collection and refinement statistics

Dataset	Hg derivative	Apo	AppCp complex	ATP complex
**Data collection(a)**				
Beamline	ESRF ID29	ESRF ID14-EH1	SOLEIL PROXIMA 1	ESRF ID29
Wavelength (Å)	0.976	0.934	0.918	0.992
Space group	P222_1_	P222_1_	P222_1_	P222_1_
Unit cell dimensions (Å)	a = 62.9; b = 73.5; c = 107.6	a = 62.9; b = 73.4; c = 106.9	a = 63.0; b = 73.5; c = 106.9	a = 63.0; b = 73.8; c = 107.4
Resolution range (Å)	54.3–1.92 (2.02–1.92)	40.7–1.47 (1.55–1.47)	35.6–1.15 (1.21–1.15)	50.0–1.71 (1.77–1.71)
Reflections (measured/unique)	193,566/38,383	916,462/84,256	708,576/173,534	256,821/54,657
Completeness (%)	98.9 (94.8)	99.9 (99.2)	98.6 (92.6)	96.8 (97.0)
Multiplicity	5.0 (5.2)	10.9 (8.6)	4.1 (2.7)	4.7 (4.8)
*R*_sym_(b)	0.065 (0.690)	0.057 (0.242)	0.026 (0.572)	0.080 (0.720)
Mean [(I)/σ (I)]	12.8 (1.9)	24.6 (7.7)	21.7 (1.8)	10.7 (1.9)
Monomers per asymmetric unit	1	1	1	1
Matthews coefficient (Å^3^ Da^−1^)	2.50	2.49	2.49	2.57
Solvent content (%)	50.9	50.5	50.7	52.1
**Refinement**				
Resolution range (Å)		40.7–1.47	35.6–1.15	47.9–1.71
*R*_factor_(c)/Free *R*_factor_(d) (%)		14.0/18.0	14.5/16.3	17.7/20.5
Unique reflections (working/test set)		80,007/4,204	173,483/8,675	54,627/2,773
Water molecules		574	612	499
Total number of atoms		4,143	4,328	4,053
r.m.s.d. bond lengths (Å)		0.014	0.012	0.007
r.m.s.d. bond angles (°)		1.41	1.03	1.04
**Ramachandran plot statistics**				
Residues in allowed regions (%)		100.0	100.0	100.0
DPI(e) (Å)		0.059	0.040	0.095

^(a)^Values in parenthesis correspond to the outermost resolution shell.

^(b)^*R*_sym_ = ∑*h*∑*i* |*Ii*(*h*)–〈*I*(*h*)〉|/∑*h*∑*i* *Ii*(*h*), where *I* is the observed intensity and 〈*I*〉 is the average intensity of multiple observations of symmetry-related reflections.

^(c)^*R*_factor_ = ∑||F_o_|–|F_c_||/∑|F_o_| where |F_o_| and |F_c_| are observed and calculated structure factor amplitudes, respectively.

^(d)^Free *R*_factor_ is the cross-validation *R*_factor_ computed for a randomly chosen subset of 5% of the total number of reflections, which were not used during refinement.

^(e)^Diffraction-data precision indicator.
