# Supplementary material for: Structure of mycobacterial maltokinase, the missing link in the essential GlgE-pathway
Source: Sci Rep. 2015 Jan 26;5:8026. doi: 10.1038/srep08026 (PMC4306142; doi:10.1038/srep08026)
Supplement: Supplementary Information — Supplementary material [file srep08026-s1.pdf]

Supplementary Materials

Structure of mycobacterial maltokinase, the missing link in the essential GlgE-pathway

Joana Fraga[a*], Ana Maranha[b*], Vitor Mendes[b], Pedro José Barbosa Pereira[a], Nuno Empadinhas[b], Sandra Macedo-Ribeiro[a]

[a] IBMC – Instituto de Biologia Molecular e Celular, Universidade do Porto, Porto, Portugal
[b] CNC - Center for Neuroscience and Cell Biology, University of Coimbra, Coimbra, Portugal

* - These authors contributed equally to this work and share first authorship.

Correspondence should be addressed to: Nuno Empadinhas (numenius@cnc.uc.pt) or Sandra Macedo-Ribeiro (sribeiro@ibmc.up.pt)

Supplementary Methods

Circular dichroism measurements

The secondary structure of Mak^{Mvan}, Mak^{Mtb} and selected point mutants was determined by far-UV CD. Measurements of samples (0.1 mg/mL in 50mM BTP pH 7.5, 50 mM NaCl) were performed at 20°C on a Jasco J-815 spectrometer set up to 1 nm bandwidth, 1 s response, 200 nm/min scanning speed and 10 accumulations. Data were analysed and deconvoluted using the DichroWeb web server[1].

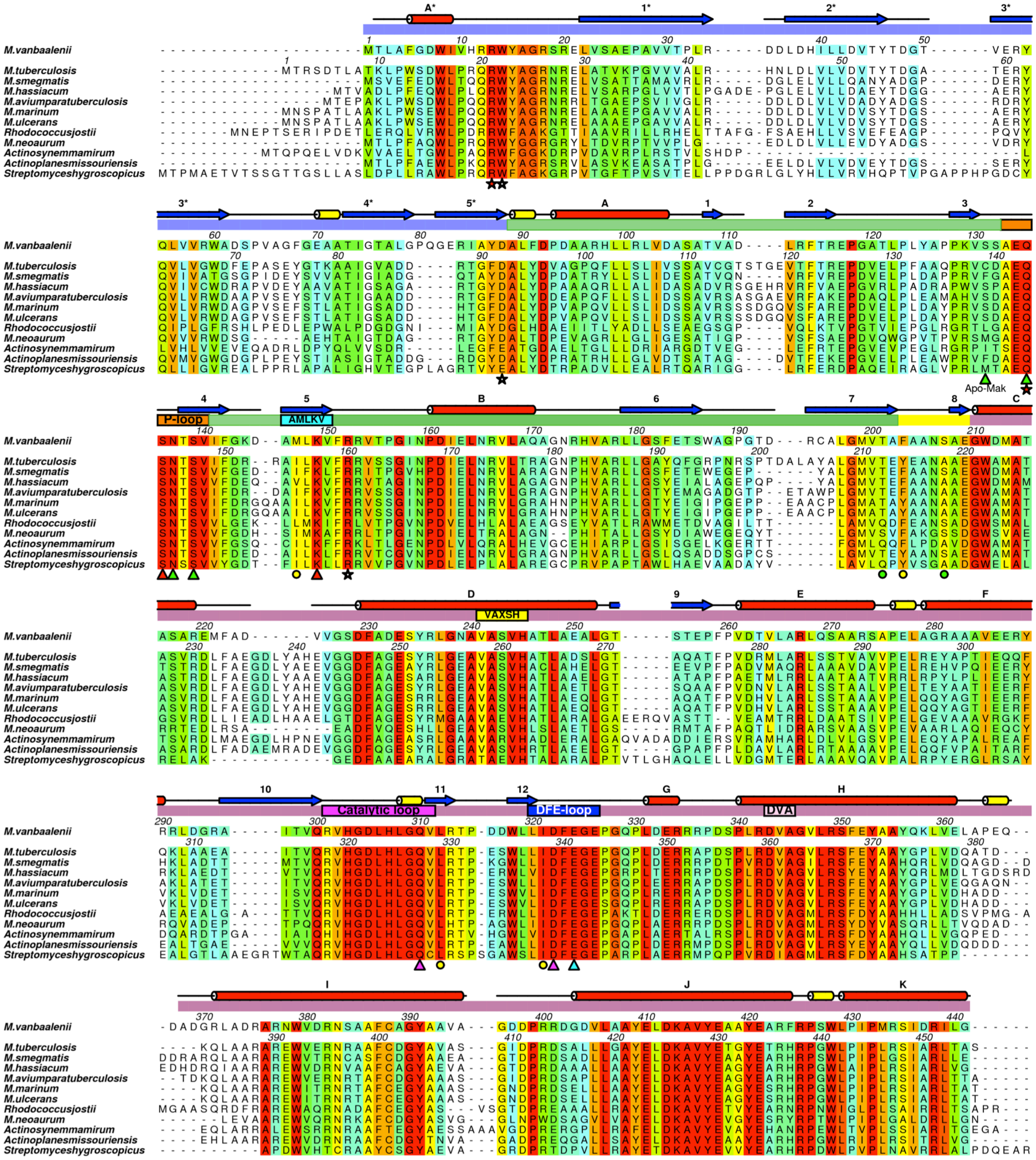

Supplementary Fig. S1 – Amino acid sequence alignment of representative maltokinases from actinobacteria

Residues are coloured according to a residue conservation scale (red: identical residues, orange to blue: scale of decreasing conservation of amino acid properties in each alignment column; white: dissimilar residues). Amino acid numbers are given above the *M. vanbaalenii* and *M. tuberculosis* sequences, and the secondary structure elements depicted above the alignment are based on the Mak^{Mvan} three-dimensional structure (red cylinders, α-helices; yellow cylinders, 3_{10}-helices; blue arrows, β-sheets). Coloured

lines above the alignment indicate the domains identified in the three-dimensional structure (blue: cap subdomain; green: intermediate domain; pink: C-terminal domain) and the main structural motifs associated with phosphotransfer activity are highlighted. Residues involved in interactions with the nucleotide are marked below the alignment (stars: residues involved in polar contacts at the interface between the cap and the intermediate domain; yellow circles: residues involved in Van der Waals interactions with the adenine base; green circles: residues involved in water-mediated interactions with the adenine base; red triangles: residues involved in direct hydrogen bonds with the γ-phosphoryl group; green triangles: residues involved in water-mediated polar contacts with the phosphoryl groups; blue triangles: residues directly stabilizing magnesium-coordinating water molecules; magenta triangles: residues involved in magnesium ion coordination). Figure prepared with Aline[2].

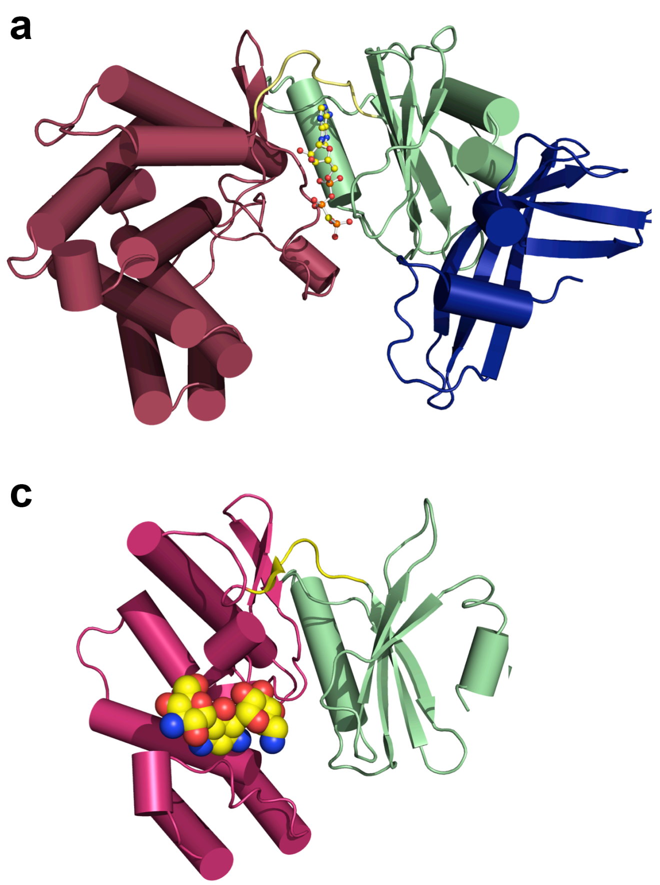

Supplementary Fig. S2 – Location of nucleotide and substrate binding sites in MAK structural homologs

(A) Mak^{Mvan}; (B) MTRK (PDB accession code 2PUN[3]; (C) aminoglycoside-3'-phosphotransferase-IIa (PDB accession code 1ND4[4]) and (D) 3',5"-aminoglycoside phosphotransferase Type IIIa (PDB accession code 3TM0[5]). Topologically equivalent domains in the different proteins are colored in a similar way, the (adenosine-5´-[(β,γ)-methyleno] triphosphate (AppCp , A and B) and adenosine 5'-[(β,γ)-imido] triphosphate (AppNp, D) analogues bound at the active site cleft are shown as ball-and-stick (A) or as sticks (B, D). The substrate and ligands bound to the substrate binding pocket in these enzymes are shown as spheres with oxygen atoms in red, nitrogen in blue, phosphorus in orange and carbon in yellow. Notice the different orientation of the γ-phosphoryl moiety in Mak^{Mvan} (A) and in MTRK (B) or aminoglycoside phosphotransferase (D).

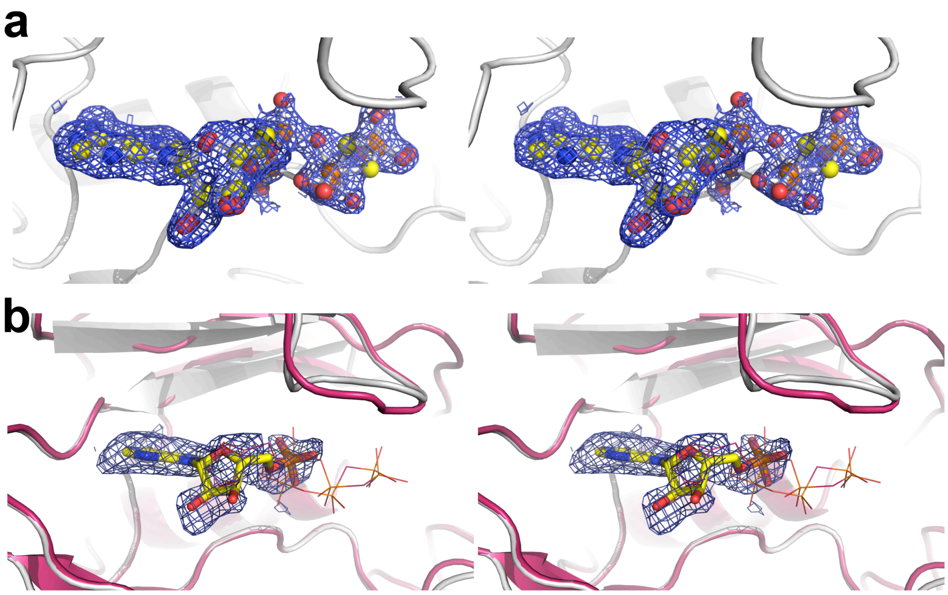

Supplementary Fig. S3 – Nucleotide binding site

(A) Alternate binding mode of AppCp to Mak^{Mvan}. Stereogram of Mak^{Mvan} active site showing the experimental 2Fo-Fc electron density map (blue mesh, contoured at 1σ), and the two modelled conformations of the bound AppCp nucleotide analogue (represented as ball-and-stick with carbon coloured yellow, nitrogen blue, oxygen red, and phosphorous orange). (B) The binding mode of ATP to Mak^{Mvan}. Stereogram of Mak^{Mvan} active site (the Mak^{Mvan}:ATP complex [white cartoon] is shown superposed to the Mak^{Mvan}:AppCp complex [pink cartoon]) showing the experimental 2Fo-Fc electron density map (blue mesh, contoured at 0.7σ) for the bound ligand (sticks, atoms color-coded as in (A)). The two identified conformations of the AppCp in the Mak^{Mvan}:AppCp complex are shown as lines, for comparison purposes. No electron density is found for the two terminal phosphates, which were removed from the final model.

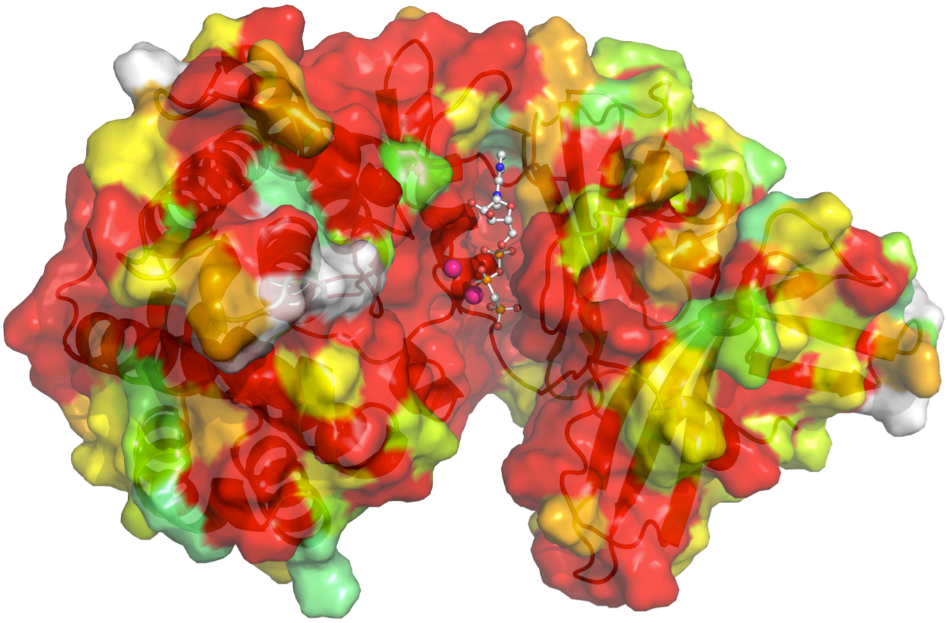

Supplementary Fig. S4. Conservation of key residues in *M .tuberculosis* maltokinase

Surface representation of Mak^{Mvan} coloured according to residue conservation between the maltokinases from *M. vanbaalenii* and *M. tuberculosis* (red: identical residues, orange to blue: scale of decreasing conservation of amino acid properties; white: dissimilar residues). Figure prepared with PyMOL (http://www.pymol.org).

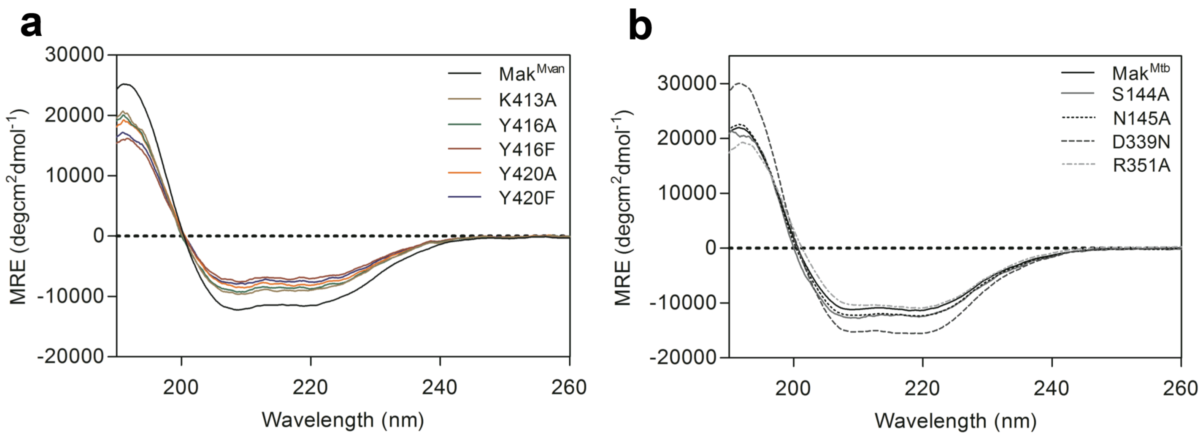

Supplementary Fig. S5 – Far UV CD spectra of Mak variants

CD spectra of wild-type Mak^{Mvan}(A) and Mak^{Mtb} (B) and of their point mutants, revealing no significant differences in secondary structure content.

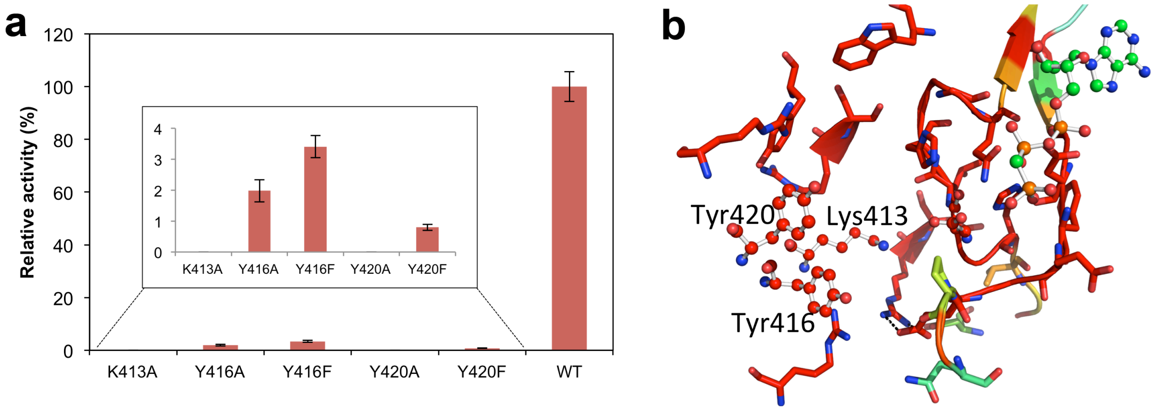

Supplementary Fig. S6 - Mutational analysis of Mak^{Mvan} maltose binding site

(A) Analysis of the effect of point mutations in key residues for Mak^{Mvan} catalytic activity. (B) Detailed view of the putative maltose-binding cavity (as in Fig. 6B) highlighting the residues selected for mutational analysis as ball-and-stick representation (oxygen atoms in red, nitrogen in blue, phosphorus in orange and carbon coloured according to sequence conservation as in Supplementary Fig. S4). AppCp is shown as ball-and-stick with carbons in green. Panel B prepared with PyMOL (http://www.pymol.org).

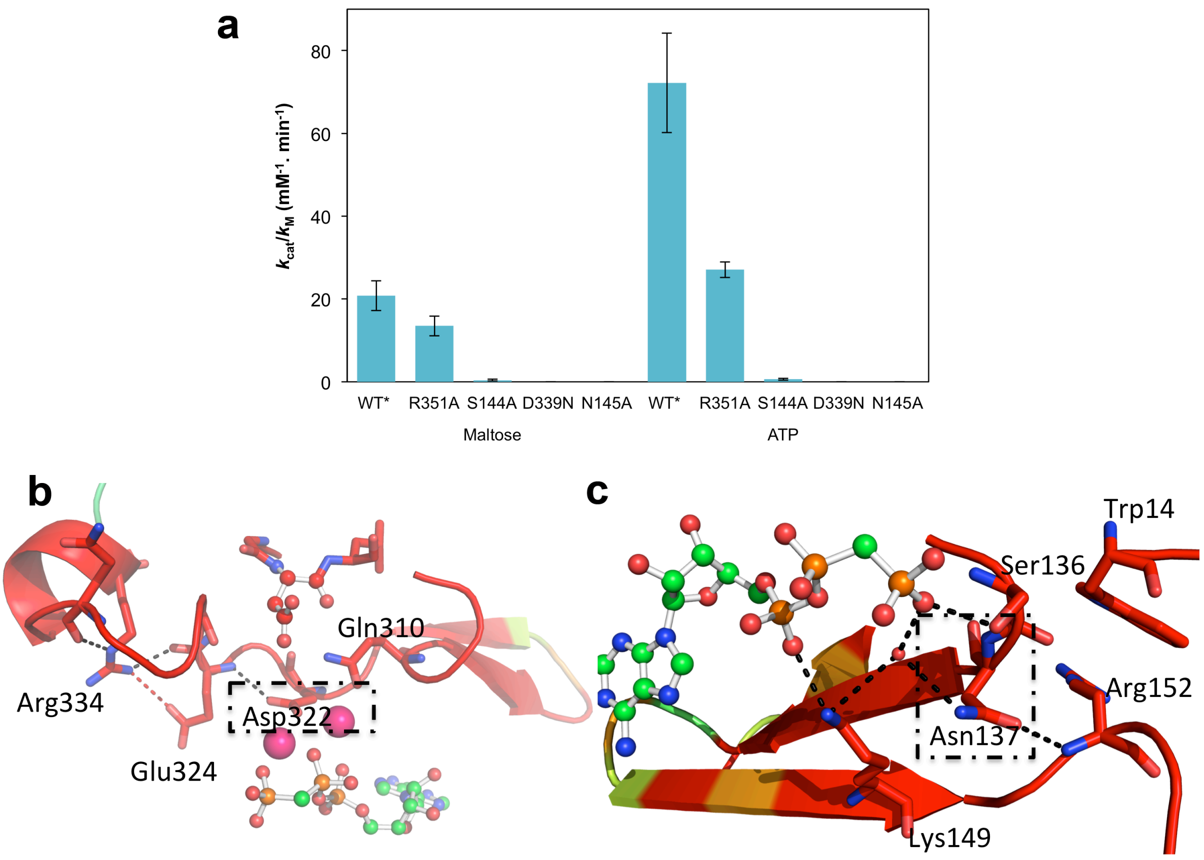

Supplementary Fig. S7 – Mutational analysis of *M. tuberculosis* Mak P-loop residues and DFE motif

(A) Analysis of the effect of point mutations in key residues for Mak^{Mtb} catalytic efficiency (k_{cat}/k_M). The mutated residues Arg351, Ser144, Asp339, and Asn145 are conserved in Mak^{Mvan} (Arg334, Ser136, Asp322, and Asn137, respectively) (B-C) Detailed view of key interactions in the catalytic loop (B) and in the P-loop (C) of Mak^{Mvan}, highlighting the residues selected for mutational analysis (dashed rectangles). Selected residues are shown as sticks and Asp305 and nucleotide as ball-and-stick with oxygen atoms in red, nitrogen in blue, phosphorus in orange and carbon in green (nucleotide) or according to sequence conservation (protein, colours as in Supplementary Fig. S4). Hydrogen bonds are represented as dashed black lines and magnesium ions as magenta spheres. Panels B and C prepared with PyMOL (http://www.pymol.org).

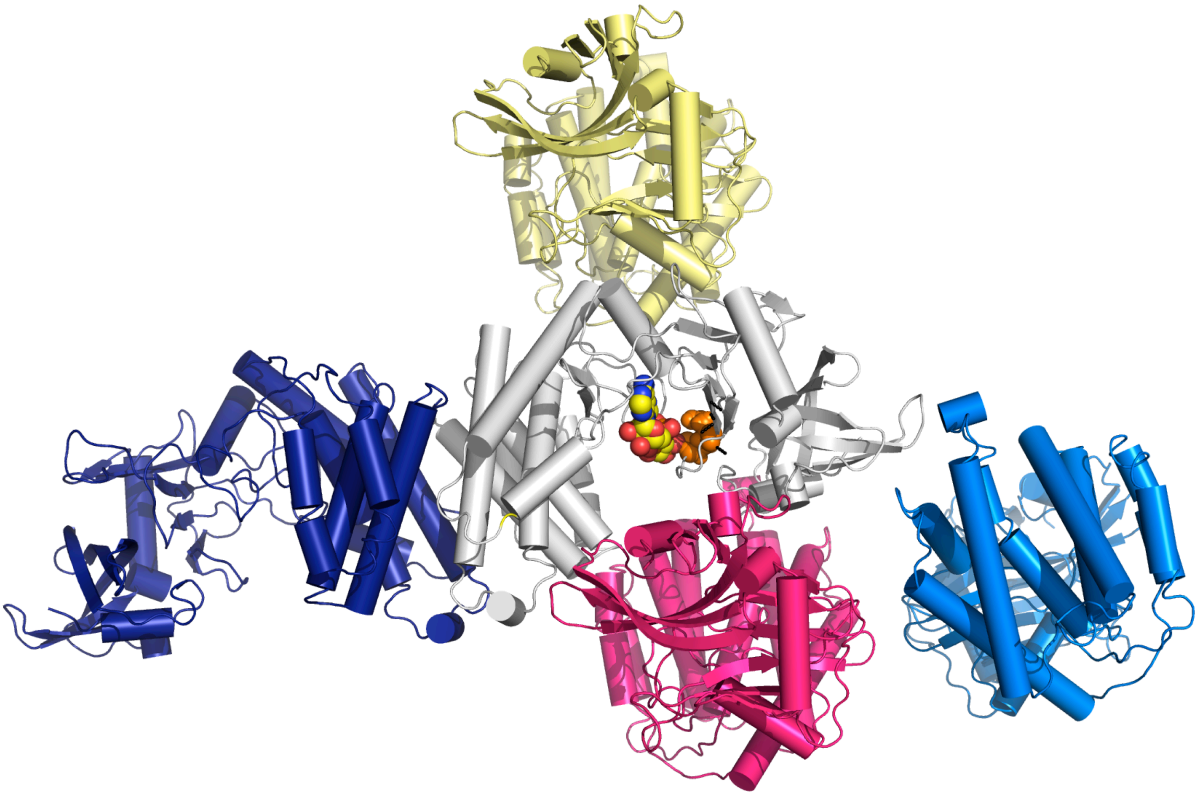

Supplementary Fig. S8 – Symmetry neighbours in Mak^{Mvan} crystals

Mak^{Mvan} is represented as a white cartoon and the nucleotide (two modelled conformations) as spheres. The P-loop residues Ser136 and Asn137 are represented as orange spheres. The neighbouring molecules in the crystal involved in significant crystal contacts are shown as cartoon representations in different colours. The largest crystal contact interface involves the C-terminal helical domain (dark blue; crystal contact buries 6% of the total solvent-accessible area), followed by a crystal contact affecting the tips of the central cleft, particularly the N-terminal domain and a large portion of the C-terminal domain (pink; buries 5% of total solvent-accessible area). The crystal contacts mediated by the external face of the intermediate (yellow) and N-terminal subdomains (marine blue) are less extensive, burying only 3% and 2% of total solvent-accessible area, respectively.

Supplementary Table S1: $\underline{Mak^{Mvan}}$ structural neighbours

Protein name	PDB entry	Z-score / r.m.s.d. (Å)	Number of aligned residues	Amino acid sequence identity* (%)
**Full length protein**				
Methylthioribose kinase	2pul[3]	19.0 / 3.7	272	10
Choline/ethanolamine kinase family protein	3dxq	18.6 / 4.1	258	13
Homoserine kinase	2ppq	18.4 / 3.3	261	15
YTAA protein	2q83[6]	18.1 / 3.3	271	16
Spectinomycin phosphotransferase	3i1a[7]	16.9 / 3.6	259	12
Putative aminoglycoside phosphotransferase	3csv	16.2 / 3.6	255	13
**N-terminal Cap domain**				
Multicystatin	2w9p[8]	4.3 / 2.6	59	14
Serine/threonine protein kinase GCN2	1zy4[9]	4.2 / 3.3	60	7
Putative NTF2-Like Transpeptidase	3k7c	4.1 / 2.7	61	10

*Structure-based sequence alignment, as calculated by Dali server (http://ekhidna.biocenter.helsinki.fi/dali_server/start)[10].

Supplementary Table S2 – Summary of parameters for pockets identified in free Mak^{Mvan}

Pocket number	Total SASA (Å^2)	Polar SASA (Å^2)	Apolar SASA (Å^2)	Volume (Å^3)	Proportion of polar atoms (%)
1	719	352	357	1728	36.6
2	591	345	246	1572	53.3
3	513	221	292	1071	45.5
4	137	48	89	209	54.2
5	377	200	177	724	46.1

SASA – Solvent-accessible surface area

Supplementary Table S3 – Oligonucleotides used as PCR primers

Code	Name	Sequence (5'-3')
MakF	MakF	ATTGATCAACATATGACGCTGGCATTCGGCGATTG
MakR	MakR	ATCAAGCTTGCCCAGGATGAGGCTGATCGATC
A	WT_NdeF	CTTACATATGACTCGGTCGGACACGC
B1	S144A_F	GACGCCGAACAGGCCAACACCAGTG
C1	S144A_R	CACTGGTGTTGGCCTGTTCGGCGTC
D	WT_HindR	ATTAAGCTTGCTAGCGGTCAGGCGGG
B2	N145A_F	GACGCCGAACAGAGCGCCACCAGTG
C2	N145A_R	CACTGGTGGCGCTCTGTTCGGCGTC
	Mak K413A_F	GCCTACGAGCTCGACGCGGCGGTGTACGAAGC
	Mak K413A_R	GCTTCGTACACCGCCGCGTCGAGCTCGTAGGC
	Mak Y416A_F	CTCGACAAGGCGGTGGCCGAAGCCGCTTACGA
	Mak Y416A_R	TCGTAAGCGGCTTCGGCCACCGCCTTGTCGAG
	Mak Y416F_F	CTCGACAAGGCGGTGTTCGAAGCCGCTTA
	Mak Y416F_R	TAAGCGGCTTCGAACACCGCCTTGTCGAG
	Mak Y420A_F	GGTGTACGAAGCCGCTGCCGAGGCCCGTTTCC
	Mak Y420A_R	GGAAACGGGCCTCGGCAGCGGCTTCGTACACC
	Mak Y420F_F	TGTACGAAGCCGCTTTCGAGGCCCGTTTC
	Mak Y420F_R	GAAACGGGCCTCGAAAGCGGCTTCGTACA

NdeI and HindIII restriction sites are underlined. Mutated codons are boxed.

1 Whitmore, L. & Wallace, B. A. DICHROWEB, an online server for protein secondary structure analyses from circular dichroism spectroscopic data. *Nucleic Acids Res* **32**, W668-673 (2004).
2 Bond, C. S. & Schuttelkopf, A. W. ALINE: a WYSIWYG protein-sequence alignment editor for publication-quality alignments. *Acta Crystallogr D Biol Crystallogr* **65**, 510-512 (2009).
3 Ku, S. Y. *et al.* Structures of 5-methylthioribose kinase reveal substrate specificity and unusual mode of nucleotide binding. *J Biol Chem* **282**, 22195-22206 (2007).
4 Kannan, N., Taylor, S. S., Zhai, Y., Venter, J. C. & Manning, G. Structural and functional diversity of the microbial kinome. *PLoS Biol* **5**, e17 (2007).
5 Nobre, A., Alarico, S., Maranha, A., Mendes, V. & Empadinhas, N. The molecular biology of mycobacterial trehalose in the quest for advanced tuberculosis therapies. *Microbiology* **160**, 1547-1570 (2014).
6 Scheeff, E. D. *et al.* Genomics, evolution, and crystal structure of a new family of bacterial spore kinases. *Proteins* **78**, 1470-1482 (2010).
7 Fong, D. H., Lemke, C. T., Hwang, J., Xiong, B. & Berghuis, A. M. Structure of the antibiotic resistance factor spectinomycin phosphotransferase from *Legionella pneumophila*. *J Biol Chem* **285**, 9545-9555 (2010).
8 Nissen, M. S. *et al.* Characterization of *Solanum tuberosum* multicystatin and its structural comparison with other cystatins. *Plant Cell* **21**, 861-875 (2009).
9 Padyana, A. K., Qiu, H., Roll-Mecak, A., Hinnebusch, A. G. & Burley, S. K. Structural basis for autoinhibition and mutational activation of eukaryotic initiation factor 2alpha protein kinase GCN2. *J Biol Chem* **280**, 29289-29299 (2005).
10 Holm, L. & Rosenstrom, P. Dali server: conservation mapping in 3D. *Nucleic Acids Res* **38**, W545-549 (2010).
